# The Huge Role of Tiny Impurities in Nanoscale Synthesis

**DOI:** 10.1021/acsnanoscienceau.3c00056

**Published:** 2024-04-08

**Authors:** Angira Roy, Ciaran P. Healey, Nathaniel E. Larm, Piyuni Ishtaweera, Maryuri Roca, Gary A. Baker

**Affiliations:** † Department of Chemistry, University of Missouri, Columbia, Missouri 65211, United States; ‡ Chemistry Department, Skidmore College, Saratoga Springs, New York 12866, United States; § Department of Chemistry, United States Naval Academy, Annapolis, Maryland 21402, United States

**Keywords:** nanocarbon, semiconductor, thermoelectric, perovskite, nanomaterial, nanoparticle, doping, contaminants, purity

## Abstract

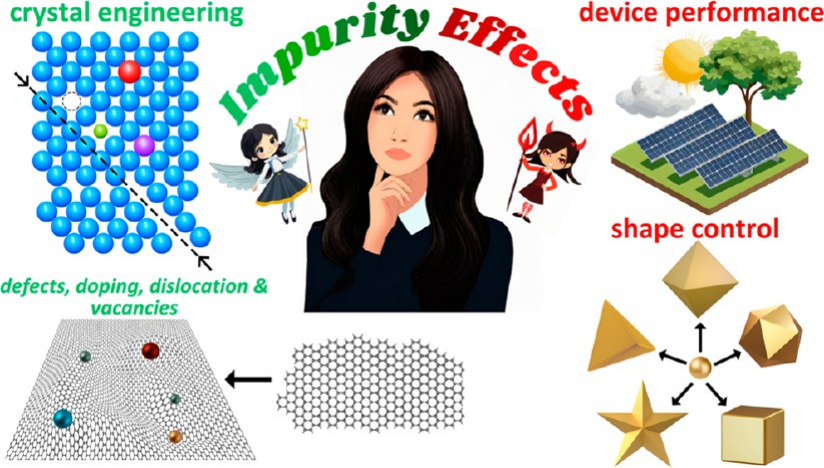

Nanotechnology is vital to many current industries, including electronics, energy, textiles, agriculture, and theranostics. Understanding the chemical mechanisms of nanomaterial synthesis has contributed to the tunability of their unique properties, although studies frequently overlook the potential impact of impurities. Impurities can show adverse effects, clouding the interpretation of results or limiting the practical utility of the nanomaterial. On the other hand, as successful doping has demonstrated, the intentional introduction of impurities can be a powerful tool for enhancing the properties of a nanomaterial. This Review examines the complex role of impurities, unintentionally or intentionally added, during nanoscale synthesis and their effects on the performance and usefulness of the most common classes of nanomaterials: nanocarbons, noble metal and metal oxide nanoparticles, semiconductor quantum dots, thermoelectrics, and perovskites.

## 1 Introduction

Nanomaterials offer novel, emergent, or amplified phenomena over their bulk counterparts due to their small size, high surface-to-volume ratio, variable morphology, and interesting quantum, optoelectronic, and magnetic properties. These nanomaterial qualities can be tuned by controlling synthesis parameters, such as energy input (e.g., temperature, light, electricity), pH, reactant concentration, reaction time, and the presence of nonreacting additives (impurities); indeed, a true vision of nanomaterials’ origin and function can only be achieved if these parameters are fully controlled and understood.

Reaction impurities can influence the synthesis and resulting properties of nanomaterials to a greater degree than one may initially anticipate. In the context of nanomaterials, “impurity” can be defined as any foreign substance or element that is present in the nanostructured material, either inadvertently from external sources or deliberately added to influence and modulate its size, morphology, and various physical and chemical characteristics. Some impurities behave beneficially and impart desirable effects on nanomaterial syntheses.^1−3^ These impurities impact the synthetic conditions, compete with the primary reaction, and direct the formation of the resulting nanomaterial.^4−10^ Conversely, even otherwise inert impurities can compromise nanomaterial synthesis and applicability.^11−14^

Impurities can originate from chemical reagents, from the environment of the synthesis, or as byproducts (i.e., secondary impurities), and limitations in achieving exceptional and reproducible batch-to-batch or vendor-to-vendor chemical purity (cost, time, and feasibility) make managing impurities an enduring problem. Rather than combat these impurities, there exists a body of research dedicated to examining their role during chemical synthesis to offer a semblance of control over, and understanding of, their impact.^15−22^ Moreover, theoretical studies are now incorporating impurities as an integral part of their analyses.^23,24^

**Figure 1:**
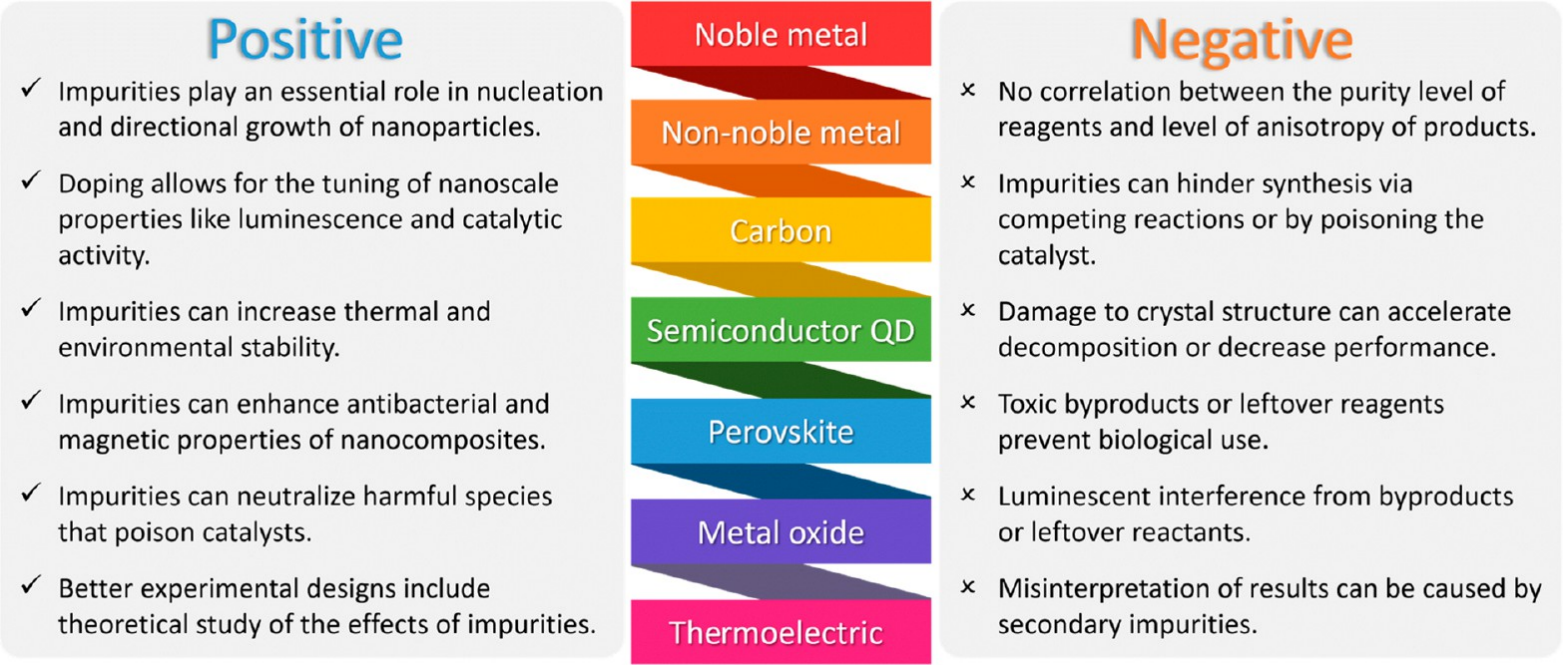
Positive and negative effects of impurities on the synthesis of nanomaterials.

In this Review, we explore the impact of impurities on nanoparticle (NP) synthesis, whether they are intentionally introduced or not. We examine works that investigate the role of purposeful impurities alongside literature reports that encountered evidence of their effects (even if the role of the impurities is not investigated or, in some cases, misattributed). Figure 1 shows some of the positive and negative effects of impurities on the synthesis of nanomaterials. While impurities can play a massive role in the synthesis and application of some nanomaterials, their impact is widely varied material-to-material. Therefore, this Review is divided for ease of reading into sections based on the prevalent nanomaterials being used today, including noble metal, non-noble metal, metal oxide, and carbon NPs, semiconductor quantum dots (QDs), and thermoelectric and perovskite nanomaterials.

## 2 Noble Metal Nanoparticles

Noble metal NPs, particularly those of Au and Ag, have captured significant attention since the groundbreaking research conducted by Michael Faraday,^25^ though their rich history spans several centuries. Initially employed in ancient Rome to add vibrant colors to glass, noble metal NPs have evolved to serve a wide range of contemporary applications in diverse fields such as optoelectronics,^26,27^ catalysis,^28,29^ sensors,^30,31^ biomedicine,^32−34^ and imaging.^31^ Some of the key factors contributing to their popularity are their nontoxic nature and the ability for researchers to precisely tune their optical, electronic, and thermal properties during synthesis. This section starts by presenting studies that encountered evidence of impurities affecting the synthesis of noble metal nanoparticles, followed by works in which impurities are explicitly considered in the experimental design and, finally, reports that highlight the role of impurities on the applicability of the nanomaterial.

**Figure 2:**
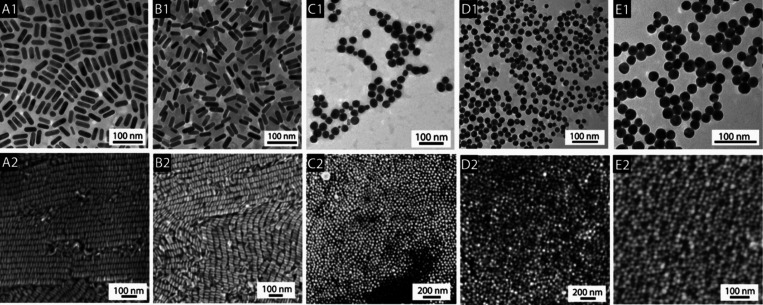
Electron microscopy images (top, TEM; bottom, SEM) of CTAB-stabilized AuNPs. The letters designate the CTAB supplier: (A) Fluka (52370, ≥96%), (B) MP Biomedicals (194004, >98%), (C) Acros (22716V, ≥99%), (D) Sigma (H5882, ≥99%), and (E) Aldrich (855820, 95%). Interestingly, sourcing CTAB from different vendors results in either rods or spheres, though the label purity does not predict the shape direction (e.g., the Aldrich CTAB is only 95% yet produced spheres). Reproduced from ref (35). Copyright 2008 American Chemical Society.

In a 2008 letter to Langmuir, Smith and Korgel demonstrated shape disparity during gold nanoparticle (AuNP) synthesis based solely on the vendor source and purity of commercial cetyltrimethylammonium bromide (CTAB), a common NP surfactant. More specifically, CTAB from Acros (≥99%), Sigma (≥99%), and Aldrich (95%) produced spherical AuNPs, whereas CTAB from Fluka (≥96%) and MPBiomedicals (>98%) resulted in Au nanorods (Figure 2).^35^ Evidently, there is significant disparity in purity between vendors, though different impurity levels can even be found lot-to-lot within the same manufacturer.^5^ The authors posit that an impurity present in some CTAB batches slows the colloidal growth rate for AuNPs, resulting in rods. Iodide in CTAB has shown similar shape control^4,18,36−38^ and can inhibit Au nanorod growth by preventing the adsorption of Au at the (111) facet, limiting elongation. Jessl and co-workers have established the correlation between the amount of iodide present in CTAB (i.e., small impurities of cetyltrimethylammonium iodide, or CTAI) and Ag^+^ concentration in Ag-assisted Au nanorod synthesis.^18^ The authors note that the exhaustive conversion of CTAI-Au^+^ to CTAI-Ag^+^ is critical to the formation of rods: CTAI-Au^+^ and CTAI-Ag^+^ exhibit similar affinities for Au (110) facets, though Ag^+^ expresses a higher reduction potential. Hence, CTAI-Ag^+^ causes the initial symmetry breaking of the seeds and drives asymmetric growth in the (100) direction. Ag^+^ also acts to prevent the unfavorable formation of CTAB-Au^+^ to increase the yield of Au nanorods, though excess Ag^+^ can result in the adsorption of CTAB-Ag^+^ on the (100) plane and reduce the rate of asymmetric growth. In another CTAB study, Liu and co-workers demonstrate the role of Ag^+^ as a shape-regulating agent to produce Au nanorods and elongated bipyramids.^39^ The authors propose that Ag^+^ selectively adsorbs to the (110) facets of Au surfaces by underpotential deposition (UPD), forming a protective monolayer of Ag^0^ while simultaneously promoting Au^0^ growth on the (111) and (100) facets. When applying single-crystalline Au seeds, this growth direction creates rods; when applying multicrystalline seeds, the products are similarly multicrystalline (bipyramids therein).

**Figure 3:**
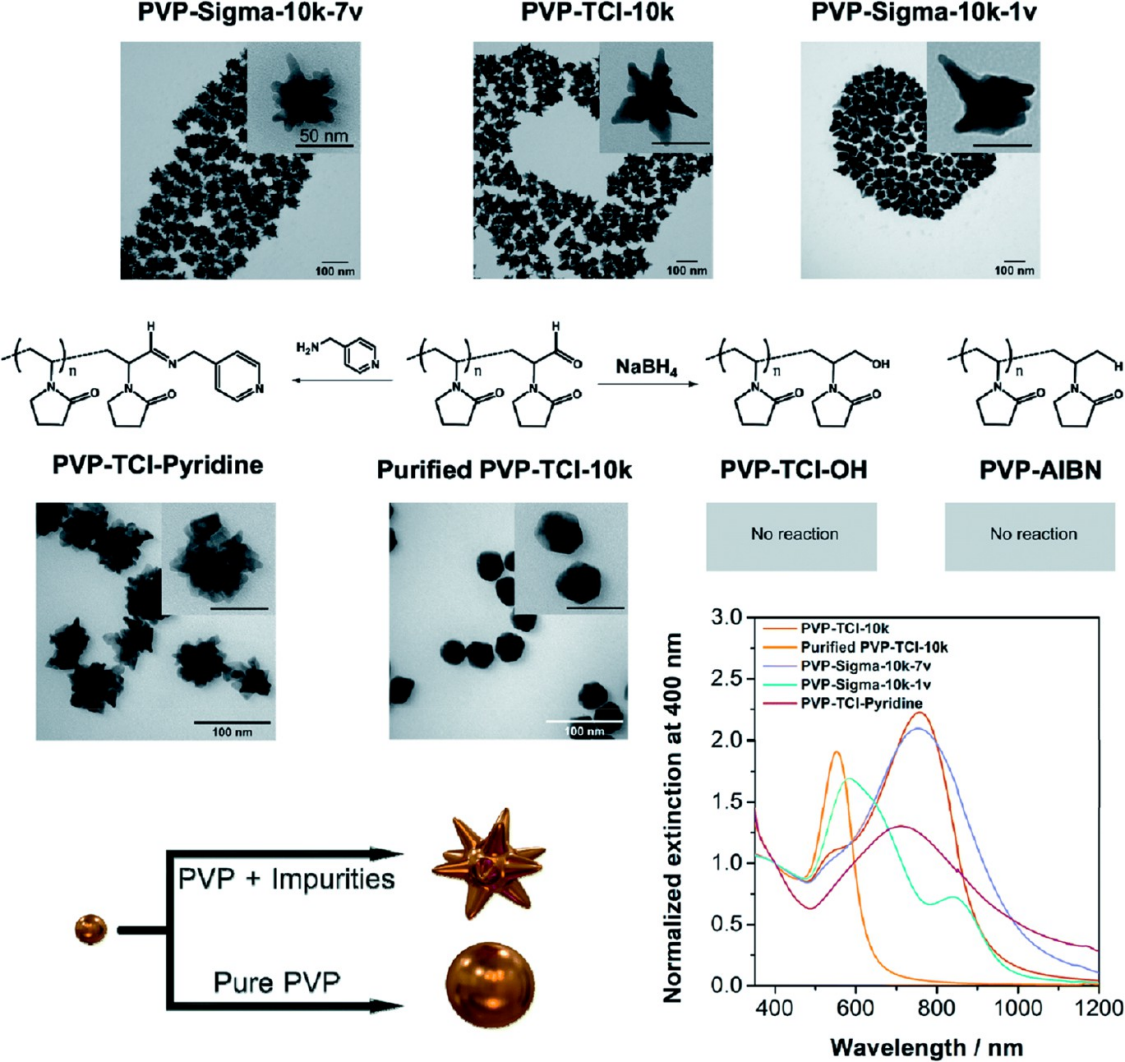
TEM images and extinction spectra of Au-NSTs synthesized using unpurified PVP, with different end groups, from two suppliers vs purified PVP. Reprinted with permission from ref (40). Copyright 2022 Royal Society of Chemistry.

Polyvinylpyrrolidone (PVP) is a popular additive during NP synthesis for metastability and to direct anisotropic growth. Silver nanoparticles (AgNPs) synthesized in the presence of purified PVP via continuous flow synthesis experience enhanced plasmonic properties independently of an added reductant, resulting in smaller particles than using unpurified PVP.^8^ However, in batch synthesis, AgNPs made using unpurified PVP exhibited much higher scattering intensity across the small angle scattering q-range because of the partial reduction of Ag in purified PVP. Similarly, the impurities in PVP can enhance the degree of anisotropy for Au nanostars (Figure 3).^40^ Taladriz-Blanco and co-workers showed another instance where disparities between supplier PVP batches resulted in wildly different Au nanostructures: in-house purified PVP resulted in Au spheres, whereas “dirty” PVP from certain vendors contained precursor impurities that shape-direct AuNPs to create nanostars. Importantly, they expanded on this discovery by recreating the nanostar formation with purified PVP by adding hydrazine. Despite the popularity of PVP as a surfactant within the research community, the identity of its impurities and their effects still needs to be deciphered.

Occasionally, impurities act as key components during NP formation. Polyvinyl acetate (PVAc) is commonly used as a NP ligand (e.g., in the radiolytic formation of AgNPs) and plays an essential role in the nucleation and growth process. However, Shin and co-workers demonstrated that purified PVAc does not produce AgNPs during radiolytic synthesis (γ-ray irradiation using ^60^Co source).^4^ Instead, AgNP synthesis only occurred when sodium acetate (NaOAc), a common impurity in PVAc, was present in sufficient quantities. Before irradiation, the acetate ion interacts with AgNO_3_, forming an Ag^+^-OAc^–^ complex which is more amenable to reduction through irradiation than the Ag^+^-NO_3_^–^ complex. Additionally, the Ag nuclei can also create an acetate complex, playing a role in promoting the growth of the nanoparticle.

The monounsaturated 18-C amine-bearing surfactant oleylamine (OAm) is frequently used for nonaqueous NP synthesis. However, the technical grade (i.e., ca. 70%) reagent contains impurities of varying chain length which can strongly influence as-synthesized NP size and shape.^41,42^ Two common impurities, elaidylamine (*trans*-9-octadeceneamine, ELAm, >15%) and octadecylamine (ODAm, <1–15%), impart opposing roles in the growth of ultranarrow Au nanowires (AuNWs).^9^ More specifically, organic syntheses of Au nanowires using specialty OAm blends result in shorter wires when doped with ODAm than with ELAm (in blends doped with both impurities, ODAm overpowers the effect of ELAm). Apart from these, other impurities in the form of octadecadienamine (5%–36%), hexadecylamine (1%–7%), and hexadecenamine are also present in substantial amounts; indeed, these impurities impact the physical properties of technical OAm and can thereby indirectly affect NP growth and stability.^43^

Ionic liquids (ILs) are increasingly common reaction media for NP synthesis and surface functionalization.^44^ They are also widely known for the controversies surrounding the impact of water, halide, acid, or parent material impurities on their physical properties. Expectedly, AgNPs produced within 1-butyl-3-methylimidazolium tetrafluoroborate ([BMIM][BF_4_]) expressed variable dispersion and agglomeration between different lots of ionic liquid solvent.^6^ Lazarus and co-workers spiked purified [BMIM][BF_4_] with water, chloride, or 1-methylimidazole and reported that contaminant levels as low as 250 ppm result in broadening and red-shifting of the plasmon band. They hypothesized that water contamination in the bulk IL interferes with the double-layer stabilization of the colloid, whereas the halide and 1-methylimidazole act as competing ligands. Accordingly, similar levels of chloride or 1-methylimidazole lead to anisotropic AgNP growth.

Impurities can be intentionally incorporated to affect the synthesis of noble metal nanoparticles. For example, controlled addition of a magnetite impurity shows that its presence during AgNPs synthesis can enhance their structural and antibacterial properties, or even integrate with the NPs to impart a magnetic response.^45,46^ Further, saturation magnetization of Ag-magnetite NPs increased by 29-fold after introducing a small concentration of cobalt nano ferrite, which makes the nanocomposite an alluring material toward various applications which can utilize these properties. Density functional theory (DFT) and atomistic models have shown that 1–2% of small impurities in the matrix of the larger atom in a binary metallic system cause a transition from a crystalline bulk-like structure to a nanocrystalline icosahedral (Ih) structure.^47^ These are especially interesting because of their enhanced catalytic and magnetic properties. But for single component clusters, face-centered cubic (fcc) truncated octahedron (TO) is more stable than Ih (i.e., for Au, Ag, and Cu). Panizon and co-workers showed that adding a small impurity, such as Cu, Co, or Ni, with these noble materials can relieve strain and induce drastic morphological changes. They studied the critical impurity percentage (I_%_) required for transforming Ih to fcc lattice and vice versa and the energy difference between the two-lattice structures at a particular I_%_.

Removing impurities has been the norm in experimental design, but purification is not always practical. Karadaghi and co-workers^48^ demonstrated the feasibility of recycling ionic liquid 1-butyl-3-methylimidazolium bis(trifluoromethylsulfonyl)imide [BMIM][NTf_2_] for the synthesis of platinum nanoparticles (PtNPs). Using the recycled solvent reduces the cost of synthesis lower than when using the 1-octadecene. In the process of synthesizing single-crystalline magnetite nanocrystals within the ionic liquid trihexyltetradecylphosphonium bis(trifluoromethylsulfonyl)imide ([P_6,6,6,14_][NTf_2_]) through an iono-polyol approach, the authors hypothesized that the enhanced uniformity observed when employing twice-recycled [P_6,6,6,14_][NTf_2_] could be attributed to the existence of residual “FeO” seeds. These seeds are believed to act as nucleation sites in subsequent reactions, leading to improved consistency in the resulting nanocrystals.^49^ Halides are common impurities in OAm and they negatively impact the controlled synthesis of AgNPs (by forming Ag halide precipitants), thus necessitating halide removal from the solvent before synthesis.^50^ In some situations, impurities can be removed successfully,^51^ but separating all the ions in other cases will compromise the quality of the nanomaterial. In another example, halides and elemental impurities in a solution of AuNPs can poison the Au catalyst. A purification approach is impractical since there are several sources for these impurities (solvent, reagents, experimental setup, drying agents, etc.). Considering the possible nature of the impurities and their affinity toward Au, Kumar and co-workers introduced a sacrificial acid into the media to neutralize the impurities and free the catalyst.^52^ Even seldomly considered wet chemistry practices like clean glassware and the quality of Milli-Q water (the trace elements and pH) used for synthesis can dramatically influence the aspect ratio of Au nanorods.^53^

Remnant reagent impurities following NP synthesis can impede their target application. For example, excess sodium citrate in AuNPs induces cytotoxicity in human alveolar cells,^54^ and free CTAB present at or above 10 μM is known to be generally toxic to humans.^55^ Schmidt and co-workers found that the robust absorption of chloride anions, originating from the reactant, in the membrane of carbon-supported Pt fuel cells during oxygen reduction reactions (ORR) can impose a kinetic constraint in the formation of H_2_O_2_.^56^ Chloride can be introduced in the preparation of the cell or as a contaminant in humidified feed streams and acts as a site-blocking species, reducing the available active sites for the ORR. Enhanced formation of peroxides reduces the stability of the membranes and causes degradation. Therefore, purified and chloride-free electrolytes and feed streams avoid performance losses.

Conversely, remnant impurities can instead enhance the properties of the nanomaterial. Formic acid oxidation (FAO) is enhanced when using a glassy carbon (GC) electrode modified with PtNPs synthesized in the presence of hydrocarbon impurities.^12^ The authors hypothesize that parts per million quantities of organic impurities (acetonitrile being prominent) disrupt CO poisoning of the Pt surface while simultaneously increasing the current peak of FAO to CO_2_. Indeed, susceptibility to CO poisoning is a known limitation of PtNPs in FAO fuel cells.^57^ These hydrocarbon impurities are common in fuel cell manufacture and extend the life of the Pt surface.^12^ Osawa and co-workers found that formic acid adsorbed on a Pt catalyst in a FAO reaction acts as a reaction intermediate instead of a site-blocking agent.^57^ Adsorbed formic acid undergoes a more rapid adsorption–desorption equilibrium than bulk formic acid and can decompose to CO_2_ and H^+^ ions. In contrast, the direct bulk oxidation pathway of formic acid to carbon dioxide is negligible,^58^ suggesting that adsorbed formic acid acts as an essential intermediate in the methanol electro-oxidation reaction.

## 3 Non-Noble Metal Nanoparticles

Transition metals are useful chemical catalysts because of their vacant d-orbitals and their ability to have variable valence states. At the nanoscale, the catalytic^59^ and electrocatalytic^60^ potential of transition metals are enhanced, and much research is focused on transition metal NPs from cheap and abundant materials like aluminum, iron, copper, and nickel.^61,62^ Because impurities cause strain in the nanocrystal, some impurities are intentionally introduced in the synthesis in order to control the catalytic power of non-noble metal NPs; however, other unintended impurities can have negative or positive effects. This section presents studies that encountered evidence of impurities affecting the synthesis, in liquid media or the gas phase, of non-noble metal nanoparticles.

**Figure 4:**
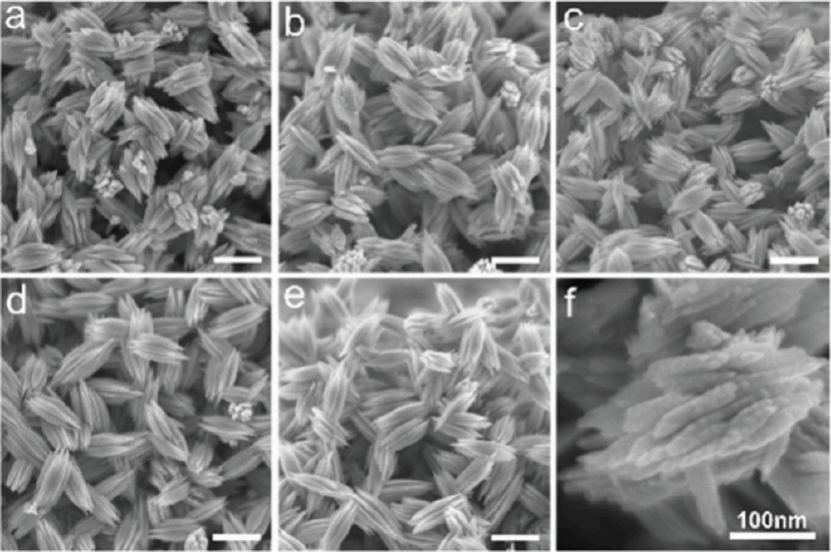
SEM images of Te particles that were obtained by mixing Te with [P_66614_]Cl IL in different polar protic alcohol solvents (a = ethanol, b = *n*-butanol, c = *n*-hexanol, d = *n*-octanol, e = *n*-decanol; scale bar for a–e is 300 nm). Panel (f) represents SEM imaging of a particle prepared in n-butanol (scale bar is 100 nm). Reprinted with permission under a Creative Commons Attribution 3.0 Unported License from ref (63). Copyright 2020 Royal Society of Chemistry.

Trace impurities in IL media used for transition metal NP syntheses affect the resulting NP morphology and play a crucial role in shape directing. Zhang and co-workers demonstrated that HCl leached from IL and the type of polar protic solvent used in synthesizing tellurium (Te) NPs affected the size and morphology of Te nano- and microstructures.^63^ The authors found that trace amounts of HCl impurities are present in trihexyltetradecylphosphonium chloride ([P_6,6,6,14_]Cl) IL and usually exist as HCl_2_^–^ anions due to a strong interaction with chloride ions. However, polar protic solvents (alcohols, water, and amines) used in the synthesis could liberate HCl from the HCl_2_^–^ anion by accelerating the cleavage of the P–Te bond in trialkylphosphanetelluride reactants, leading to the formation of highly homogeneous 3D Te fusiform assemblies and 3D aloe-like Te microarchitectures. SEM images in Figure 4 show the construction of fusiform Te structures in the presence of different polar protic solvents that are homogeneous. It has also been reported that a Brønsted acid impurity in the [P_6,6,6,14_]Cl IL leads to the surface activation of elemental copper. This is vital to obtain a quantitative product yield, leading to the formation of micrometer-sized Cu_3–*x*_P particles. According to these findings, trace impurities in ILs affect the quality of NPs synthesized therein and, with careful analysis and control, can be used as a synthetic tool.^64^

**Figure 5:**
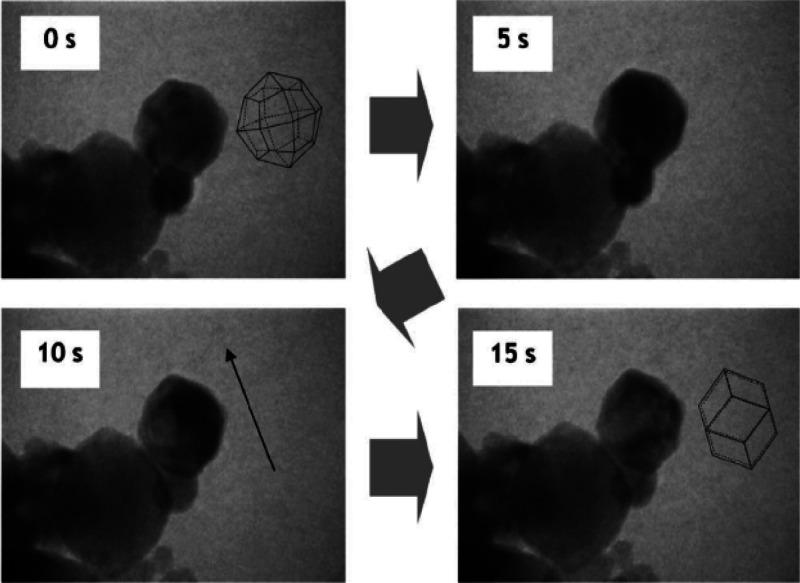
Images of the morphology transition of α-CrNPs from a rhombic dodecahedron to a cubic one upon incorporating an oxygen impurity. The shape was elongated along the direction shown by the arrow (bottom left panel). Adapted with permission from ref (65). Copyright 2005 Elsevier.

**Figure 6:**
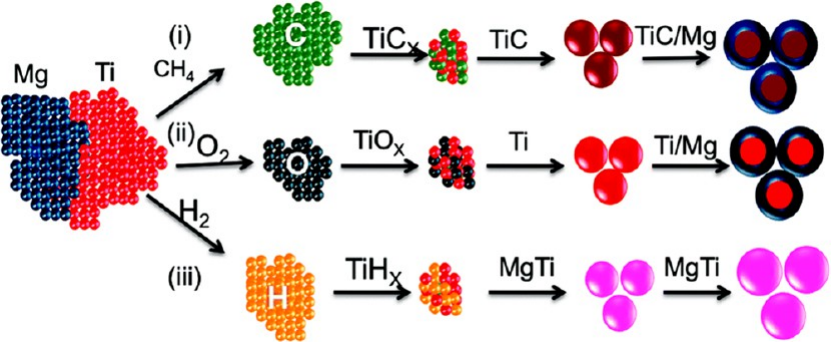
Schematic representation of the nucleation and growth of MgTiNPs. Gas impurities of CH_4_, O_2_, or H_2_ result in core/shell TiC/Mg NPs, core/shell TiO_2_ NPs, or MgTiNPs as depicted by routes (i), (ii), or (iii), respectively. Adapted with permission from ref (70). Copyright 2017 Royal Society of Chemistry.

Kido and co-workers reported that incorporating oxygen impurities into the structure of chromium nanoparticles (CrNPs) leads to a phase transition from δ-CrNPs to metastable cubic α-CrNPs (Figure 5).^65^ The authors estimated the oxygen volume to be 20–30% of the volume of the Cr_2_O_3_ surface layer. Kimoto and Nishida also showed that oxygen content in introduced gas is essential for the growth of cubic particles. However, interstitial oxygen in the cubic α-Cr particles can also act as an impurity and alter the growth.^66^ Trace amount of reactive gas is essential for nucleation for Ti, Co, and W NPs^67,68^ when homogeneous nucleation is challenging to initiate. Similarly, exposure to oxygen can alter (and often widen) the shape and size distributions of some metal NPs. For example, magnesium nanoparticles (MgNPs) form a MgO shell and exhibit a broader size distribution than Cu, Co, Fe, Nb, and Mo.^69^ The MgO formed at the Mg/MgO interface can also lead to hollow, sharp facet voids due to the different diffusion rates of Mg and oxygen. The presence of oxygen or water can be problematic for the homogeneous nucleation of these alkaline earth metals due to their lower reduction potentials. For the bimetallic gas-phase synthesis of MgTi, the introduction of trace amounts of H_2_ or CH_4_ to the reaction directs the growth of resulting NPs.^70,71^ Indeed, the presence of H_2_ directs MgTi NPs toward hexagonal platelets whereas CH_4_ directs the growth toward trigonal platelets. The authors posit that a strong Ti–C affinity results in a TiC core in the presence of CH_4_, leading to the formation of TiC/Mg core–shell NPs. On the other hand, TiH_*x*_ forms in the presence of trace H_2_, leading to the development of a MgTi solid solution (Figure 6).

## 4 Carbon Nanomaterials

Carbon is an indispensable element of organic chemistry and life, and the invention of Buckminsterfullerene^72^ opened the door of carbon nanomaterials to the world. Graphene and carbon nanotubes (CNTs)^73^ have since become the most critical structures discovered, while fluorescent carbon dots (FCDs)^74,75^ are the newest addition to this family. Carbon-based nanomaterials have gained significant attention because of their extraordinary electrical and thermal conductivities, mechanical strength, high thermal stability, low toxicity, good biocompatibility, and synthetic tunability.^76−78^ They have already been successfully used for drug and gene delivery,^79^ bioimaging,^80^ energy storage,^81^ electrochemical sensors,^82^ and even dye-sensitized solar cells.^83^ Recent studies have revealed the role of impurities in the synthesis and performance of these carbon nanomaterials. This section starts by presenting studies that encountered evidence of impurities affecting the synthesis of carbon nanomaterials, followed by works in which impurities are explicitly considered in the experimental design and, finally, reports that highlight the role of impurities on the applicability of the nanomaterial.

Metal catalysts used in synthesizing carbon nanomaterials retain impurities that enhance or inhibit electrocatalytic processes.^2^ For example, Mazánek and co-workers reported that the electrocatalytic activity at elevated temperature was almost negligible in ultrapure graphene prepared by the chemical reduction of graphene oxide. At the same time, the rate of heterogeneous electron transfer was slightly slowed down.^19^ It is crucial to enhance the mobility of graphene for electronic and sensor applications, but Coulomb impurities^84^ present in graphene affect the intrinsic electronic properties. The long-range scattering by charged impurities and short-range scattering by atomic impurities degrade the mobility and transport properties. Martin and co-workers used a Monte Carlo simulation to show that the effects of the impurities also depend on the substrate. Pure graphene on hexagonal boron nitride (h-BN) exhibits the best performance. In contrast, in the presence of Coulomb impurities, graphene on SiC shows the best results in diffusion coefficient, drift velocity, and low field mobility due to better screening of such impurities.^85^

Impurities can directly affect surface defects, such as wrinkles, cracks, and microstructural defects (MSD), thus playing an essential role in determining the crystal quality and electronic properties of graphene. Zhang and co-workers described how the growth mechanism of an MSD depends on the impurities present on the Cu substrate surface, such as Cu particles and silicon dioxide particles introduced during the chemical vapor deposition (CVD) growth of graphene.^86^ Nucleation initiates from the surface impurities on the substrate, and finally, MSDs form around those impurities. Carbon impurities on the substrate surface can damage the graphene, decrease stability, and direct nucleation, depending on the etching environment. In a weak etching growth environment, the carbon impurities act as the primary nucleus for graphene growth, while in a robust etching atmosphere, they inhibit nucleation.^16^ Zang and Li reported that isotope impurity in single-walled carbon nanotubes could reduce conductance by 60% and change temperature-dependent behaviors.^87^

**Figure 7:**
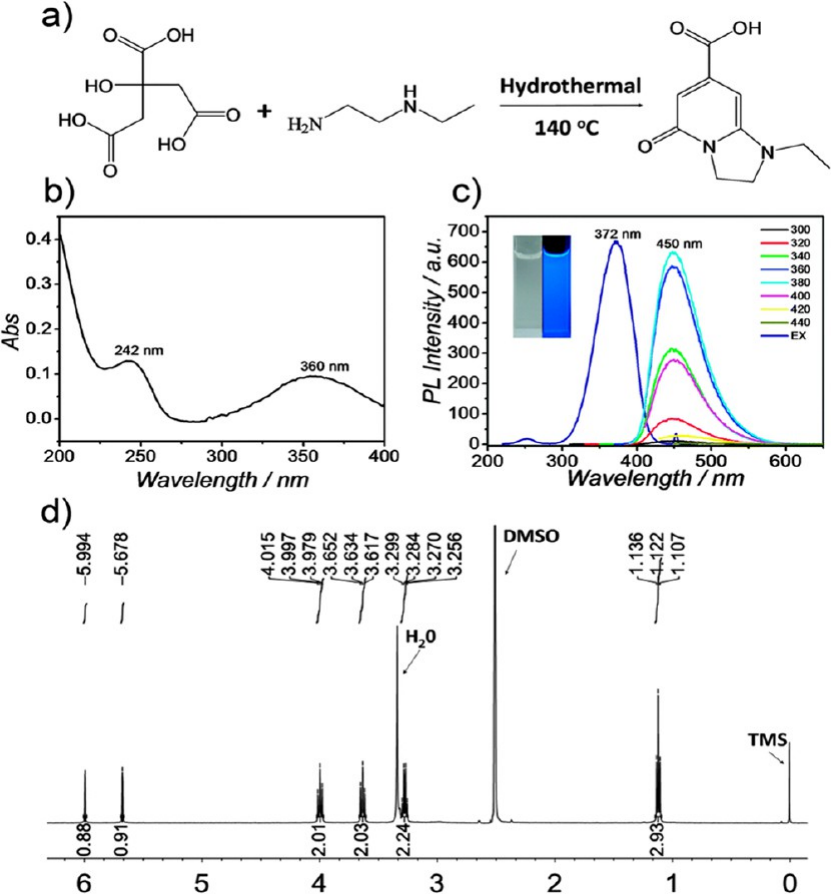
(a) Schematic synthesis of PL Et-CDs using citric acid and *N*-ethylmethane-1,2-diamine (Et-EDA). Panels (b)–(d) represent characterization of these CDs: (b) UV–vis absorption spectrum, (c) PL spectra (with inset photographs of Et-CDs solution under ambient and UV illumination), and (d) ^1^H NMR spectrum of 1-ethyl-5-oxo-1,2,3,5-tetrahydroimidazo[1,2-*a*]pyridine-7-carboxylic acid (Et-IPCA), a product of the hydrothermal reaction of CA and Et-EDA separated from Et-CDs). Adapted with permission from ref (89). Copyright 2015 Royal Society of Chemistry.

Impurities often lead to misinterpretation and confusion in the practical implementation of carbon materials, as specific impurities and concentrations vary from sample to sample. One example of this principle is in CNT sensors; because a significant deviation has been observed in electrochemical responses in similar sensors, Jones and co-workers used CNTs from Phoenix Nano-Systems, synthesized with metal-free catalysts,^88^ to avoid impurity-related misinterpretation. Further misinterpretation can be caused by secondary impurities, which originate from the synthesis reaction or reaction media, if these impurities affect the properties of the nanomaterial. For example, FCDs have gained attention due to their unique tunable fluorescence and high photostability. Still, recent reports are showing that the origin of fluorescence may be due to the organic byproduct, imidazo[1,2-*a*] pyridine-7-carboxylic acid, 1,2,3,5-tetrahydro-5-oxo- (IPCA) formed alongside FCD (Figure 7).^89^ It is clear that IPCA is the true source of Et-CDs fluorescence, synthesized using *N*-ethylmethane-1,2-diamine (Et-EDA) and citric acid. Essner and co-workers described a purification procedure of unfractionated samples by membrane dialysis^90^ to revisit the misconception about FCD photophysical properties and fluorescent applications (bioimaging, sensing, and heavy metal detection).

As purification may not always be practical, researchers must explore other creative ways to deal with impurities. Graphene conductivity depends on charged impurities.^91^ To reduce the effect of the charged impurities, Chen, Xia, and Tao introduced the ionic screening method using NaF as ion sources, as both the Na^+^ and F^–^ ions are chemically inert toward adsorption onto the graphene surface and neutralize charged impurities.^92^

Remnant impurities following synthesis can enhance or prevent their target application. Recently, the enhanced electrochemical properties of graphene/polylactic acid (PLA) 3D-printing filament have been related to the presence of Fe, Ti, and Al impurities.^14^ While metal impurities have shown both positive and negative effects on the electrocatalytic performance of carbon nanomaterials, their toxicity makes them detrimental to biomedical applications.^93^ These metal impurities severely affect cell viability due to the excessive production of reactive oxygen species (ROS) in the A549 cell line.^11^

## 5 Semiconductor Nanomaterials

The modern study of nanoscience, nanotechnology, and nanobiotechnology would not be possible without the enormous contribution of semiconductor QDs and their access to the quantum confinement effect,^94,95^ as recognized by a recent Nobel prize.^96^ Since their inception, significant advances have been achieved in synthetic procedures for QDs to control their optical, magnetic, and electronic properties.^97^ This understanding has strengthened the backbone of the nanoworld, bringing chemistry, physics, biology, and technology together and allowing for the harmonization of QDs with inorganic, organic, and biomolecules. In addition, the unique properties of QDs have extended their applications in optoelectronics, photovoltaics, telecommunication, spintronics, quantum computing, biosensing, and bioimaging.^98−101^ Many of these advances derive from the intentional incorporation of impurities in the crystal structure (doping) to tune the photophysical properties of QDs.^102^ This section presents studies that encountered or sought evidence of impurities affecting the morphology of semiconductor nanomaterials and their photophysical properties.

Since the first report of CdSe light emitting diode (LED) nanocrystals by Alivisatos and co-workers,^103^ much work has been dedicated to their improvement using impurities.^104^ The growth, morphology, and optical properties of QDs can be readily influenced by added impurities from impure reagents or doping.^105^ The growth kinetics and morphology of CdSe QDs depend on the supplier of tri-n octylphosphine oxide (TOPO), commonly used as a ligand in nanocrystal synthesis.^1,106−108^ Technical grade TOPO contains alkylphosphonic and phosphinic acid of various chain lengths, and is reported to include at least ten phosphorus-containing impurities.^109,110^ These impurities bind strongly to cadmium ions and lead to rod formation instead of a CdSe spheres.^111,112^ Wang, Tang, and Buhro probed how common impurities (e.g., dinoctylphosphine oxide, DOPO; di-*n*-octylphosphinic acid, DOPA; mono-*n* octylphosphinic acid, MOPA; and *n*-octylphosphinic acid, OPA) present in commercially available TOPO assist in nearly isotropic QD formation.^1^ DOPA, MOPA, and OPA regulate the generation of quantum rods (QRs), with DOPA promoting quantum wire (QWs) growth. The phosphonic acids play an even more prominent role depending on their chain length; short-chain phosphonic acids preferentially bind to the CdSe or CdTe facets with densest electron deficient surface site packing (i.e., {111}_ZB_ facets of zinc-blend (ZB) lattice structure and {001}_W_ facets of wurtzite (W) lattice structure). As {111}_ZB_ facets outnumber {001}_W_, the facet-to-surface energy ratio of the ZB nanocrystal is higher than that of the wurtzite nanocrystal. As a result, short-chain alkylphosphonic acid favors ZB phase of CdSe and CdTe nanocrystals, whereas long-chain phosphonic acids favor the wurtzite phase.^111,113^ Even high-purity commercially available TOPO can be decomposed into DOPA, MOPA, and OPA at elevated temperatures in the presence of residual oxygen in the reaction mixture, leading to the formation of QRs.^107^

**Figure 8:**
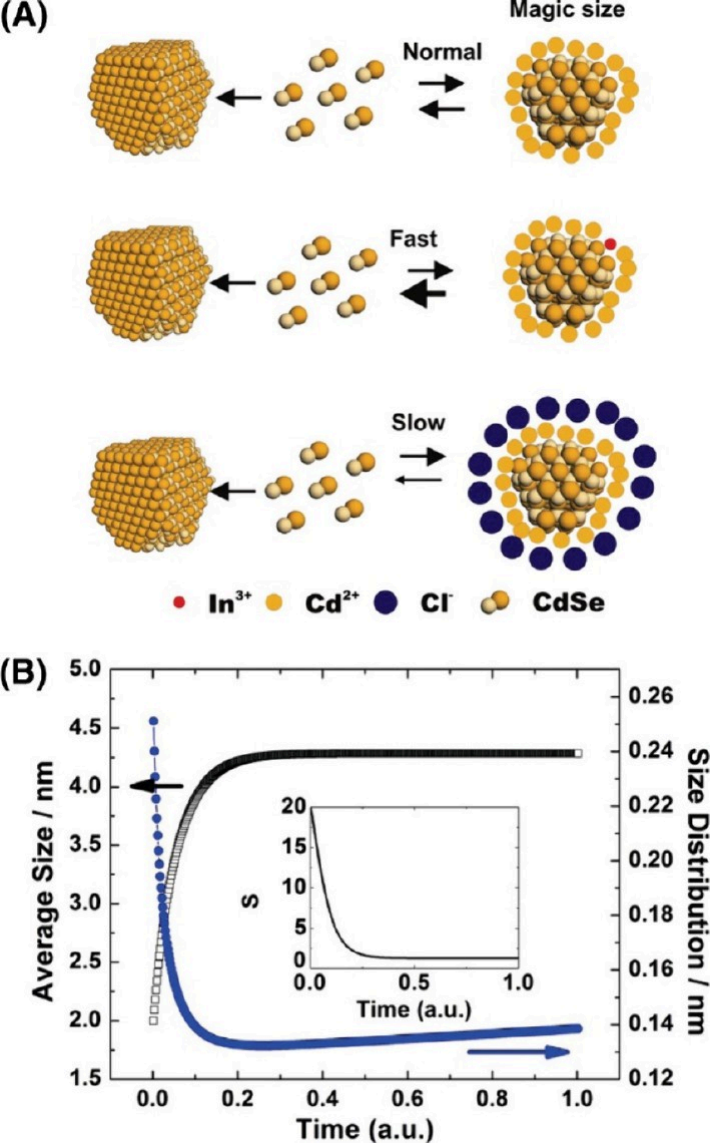
Proposed kinetic model of heterogeneous growth without adding any impurities establishes an equilibrium between the dissolution of the magic-size cluster and the free monomers in the solution. Adding In to the particle surface increases the dissolution rate of magic-size clusters to provide monomers for growth, while chloride ions stabilize the magic-size cluster against dissolution. Reproduced from ref (116). Copyright 2008 American Chemical Society.

Another popular solvent for colloidal CdSe nanocrystal synthesis is octadecene (ODE), though it forms poly(ODE) impurities at temperatures above 120 °C which inhibit charge transfer in resulting nanocrystal films.^114^ Alternative solvents, like n-hexadecane and n-octadecane, can be used to avoid these problems, though some researchers opt to instead separate the nanocrystal from poly(ODE) by exchanging it with a polar capping agent [such as (6-[2-[2-(2-methoxy-ethoxy)-ethoxy]-ethoxy] hexyl) phosphonic acid]. In these cases, the now polar nanocrystals can be separated from poly(ODE) by precipitation with the addition of hexane. Similarly, the growth kinetics of QDs can be affected by intentionally introducing impurities in the crystal, altering the particle growth and dissolution rate. Tuinenga and co-workers reported that In^3+^ impurities promote larger CdSe NP growth (Figure 8) by increasing the conversion of magic-size nanoclusters (MSNCs)^115^ to monomers.^116^ Also, the surface energy changes as +2 charged Cd is replaced by +3 charged In. This enhances the attraction of monomers to the particle surface above the rate of monomer desorption.

As previously mentioned, commercially accessible OAm is found to have notable impurities, including the trans isomer, shorter and unsaturated amines, and various luminescent components (e.g., complexes of nitroalkanes and aminoalkanes).^117^ Indeed, these impurities alter the solvent properties of the OAm and provide a barrier for luminescence-sensitive characterization techniques like Raman spectroscopy. Purification techniques must be included before or after synthesis to remove luminescent impurities that can obscure the optical properties of the nanomaterial. Baranov and co-workers found that purified OAm allows selective precipitation of PbS quantum dots before remnant lead chloride starting materials during nanocrystal washing.^118^ OAm impurities can also complicate thermal treatments. For example, Sperry and Luscombe identified a pyrolyzed graphene oxide fine-grain layer (FGL) underneath a layer of synthesized Cu_2_ZnSnS_4_ (CZTS) nanocrystals following air-free synthesis and annealing (225 °C) in OAm medium.^119^

Anionic impurities from the precursor metal salts can alter the properties of metal chalcogenide NPs. Metal acetylacetonate, halide, acetate, oxide, nitrate, hydrate, sulfates, and mixed salts are the most commonly used salts for the synthesis of Cu(In,Ga)S_2_ (CIGS), Cu_2_ZnSnS_4_ (CZTS), Cu(In,Ga)(S,Se)_2_ (CIGSSe), and Cu_2_ZnSn(S,Se)_4_, (CZTSSe) NPs. Anionic impurities, such as chlorides, can behave as n-type impurities in the device.^120^ While this problem can be avoided for CIGSSe using acetylacetonate salts, indium acetylacetonate and acetate form indium oxide due to heating in the absence of a sulfur source.^121^ Formation of such oxides can drastically obstruct the performance of photovoltaic devices. This can be particularly problematic for highly stable and high bandgap oxides like gallium oxide.^122^ This oxide can behave as an insulating layer, significantly hindering the solar cell performance. Depositing CIGS on aluminum-doped zinc oxide (AZO) by the low-temperature pulsed electron deposition technique (LTPED)^123^ can prevent the formation of Ga_2_O_3_. The LTPED approach allows for high-quality CIGS layer formation on various surfaces and substrates. To avoid the problems arising from the anionic contaminant, Deshmukh and co-workers introduced a new pathway for metal chalcogenide NPs by combining an amine-thiol-based precursor approach and colloidal NP synthesis.^124^ They dissolved the pure metals in 1,2-ethanedithiol in the desired amount to make binary, ternary, and quaternary metal thiolate precursors free from the previously mentioned anionic precursors.

**Figure 9:**
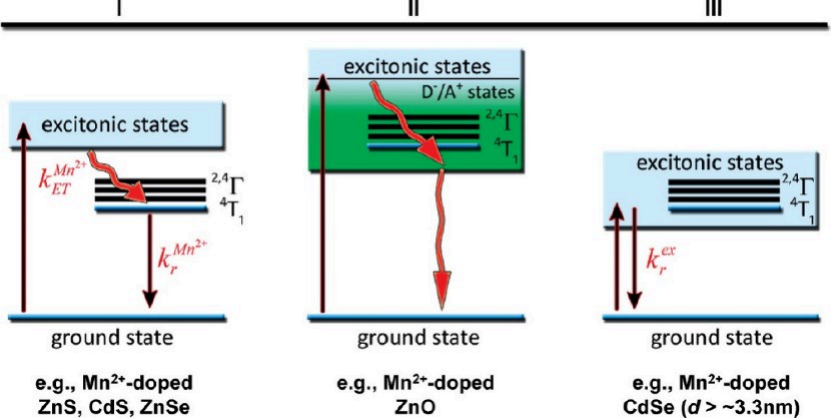
Schematic representation of different electronic structures related to photoluminescence is observed in colloidal Mn^2+^-doped semiconductor nanocrystals. In the first scenario (I), efficient energy transfer (k_ET_^Mn2+^) quenches excitonic emission and sensitizes Mn^2+^^6^A_1_ ← ^4^T_1_ luminescence. In scenario (II), excitons are quenched by Mn^2+^ photoionization states (dopant-bound excitons) that relax nonradiatively to the ground state. Finally, in scenario (III), all Mn^2+^ excited states are located outside the bandgap, and the nanocrystals show excitonic emission. Examples of Mn^2+^-doped II–VI diluted magnetic semiconductor (DMS) nanocrystals of each type are provided. Reproduced from ref (126). Copyright 2008 American Chemical Society.

Transition metal doping in QDs imparts new properties, such as thermal and environmental stability and Stokes shifts, which help to avoid self-quenching and extend excited-state lifetimes. Indeed, Mn^2+^ and Cu^2+^ are among the most common transition metal impurities imposed in QDs. For Mn^2+^-doped QDs, the altered optical properties depend on the relative position of the Mn^2+^ energy levels; in CdS, ZnS, and ZnSe hosts, the Mn^2+^ ligand field excited state lies within the bandgap of the semiconductor nanocrystal. Eilers and co-workers reported that increasing Mn^2+^ concentration in a ZnTe host decreases the excitonic luminescence intensity (∼430 nm) and increases the Mn^2+^^6^A_1_ ← ^4^T_1_ ligand field transition intensity (∼560 nm).^125^ This observation was attributed to semiconductor-to-Mn energy transfer. Also, the lifetime of the doped semiconductor was enhanced up to 200 μs because of the spin-forbidden Mn^2+^^6^A_1_ ← ^4^T_1_ transition (Figure 9). Beaulac and co-workers observed that both the bandgap and the relative position of the Mn^2+^ energy levels in CdSe changed with nanocrystal size.^126^ For smaller QDs with higher bandgaps, the Mn energy levels lie within the bandgap and exhibit two luminescence peaks; one comes from CdSe exciton emission and another due to the ^6^A_1_ ← ^4^T_1_ transition (Figure 9). Only one photoluminescence (PL) peak is observed in larger nanocrystals, as Mn energy levels lie above the host bandgap. In the case of Mn^2+^ doped ZnO QDs, the PL intensity decreases with increasing dopant concentration. Additionally, no additional peak arises due to the d-d transition in the Mn center. The presence of a sub-bandgap photoionization state leads to nonradiative relaxation back to the ground state (Figure 9). Sivasankar and co-workers reported that the optical bandgap of CdSe nanocrystals increases with the concentration of dopant Cu^2+^ ion.^127^ They proposed incorporating copper ions and creating defect levels in the bandgap to increase the carrier concentration. Cu-doped CdSe exhibits room-temperature ferromagnetism, as the localized magnetic ions in semiconductors give rise to an exchange interaction that increases the bandgap between the s-p electron of the host and the Cu d-electron. The extent of the magnetic moment increases with the Cu concentration and reaches the optimum value for 8% Cu-doped CdSe. Jabeen and co-workers reported that noble metal-doped ZnS QDs show redshift in their absorption spectra.^128^ Au-doped ZnS exhibits green light emission along with blue light, but the crystallinity of the doped ZnS decreased compared to the pure ZnS. Rekha and co-workers^129^ reported reduced photocatalytic activity when ZnO is doped with Mn.

## 6 Perovskite

In spite of their short history,^130,131^ perovskites nanomaterials are currently an exceedingly popular research area, with their tunable bandgap,^132^ large absorption cross sections,^133^ negligible electron–phonon coupling,^134^ narrow emission line width, and simple and cost-effective solution synthesis garnering widespread attention. However, perovskite nanomaterials are unstable and intolerant of harsh conditions, limiting their commercial use.^135^ Similar to semiconductor QDs, doping has become quite common to tune the properties of perovskites, though recent works have shown that some impurities can accelerate their degradation.^136,137^ This section presents studies that encountered or sought evidence of impurities affecting, positively or negatively, the synthesis of perovskite nanomaterials, followed by works in which impurities are explicitly considered in the experimental design.

Zhao and co-workers reported that doping alkali metals, such as K^+^ or Na^+^, can improve grain size, increase luminescence intensity, increase the photoluminescence quantum yield (PLQY), and decrease the carrier lifetime trap state.^138^ Because fewer trap states are equivalent to less nonradiative recombination, doping the alkaline earth metal Sr^2+^ in an organic–inorganic hybrid perovskite, methylammonium lead iodide (MAPbI_3_), can decrease the bandgap and increase thermal stability.^139^ A similar effect can be observed by replacing MA^+^ with Cs^+^ in MAPbBr_3_ nanocrystals due to lattice shrinkage and reduced defect density.^140^ Li and co-workers dug deep into this to understand the reason behind such improved chemical, thermal, and optoelectronic properties. X-ray photoelectron spectroscopy (XPS) measurements have shown that the binding energies of Br, Pb, and N (from the methylammonium cation) atoms increase monotonically with increasing Cs^+^ dopant concentration. This phenomenon indicates that Cs^+^, an isoelectric impurity, impacts atomic interaction as Cs^+^ is more electronegative than MA^+^. Thus, Cs^+^ impurities present in the Cs_*x*_MA_(1-*x*)_PbBr_3_ lattice enhance stability, lead to charge polarization, and can increase the photocurrent in Cs_*x*_MA_(1-*x*)_PbBr_3_ photodetectors.^141^

**Figure 10:**
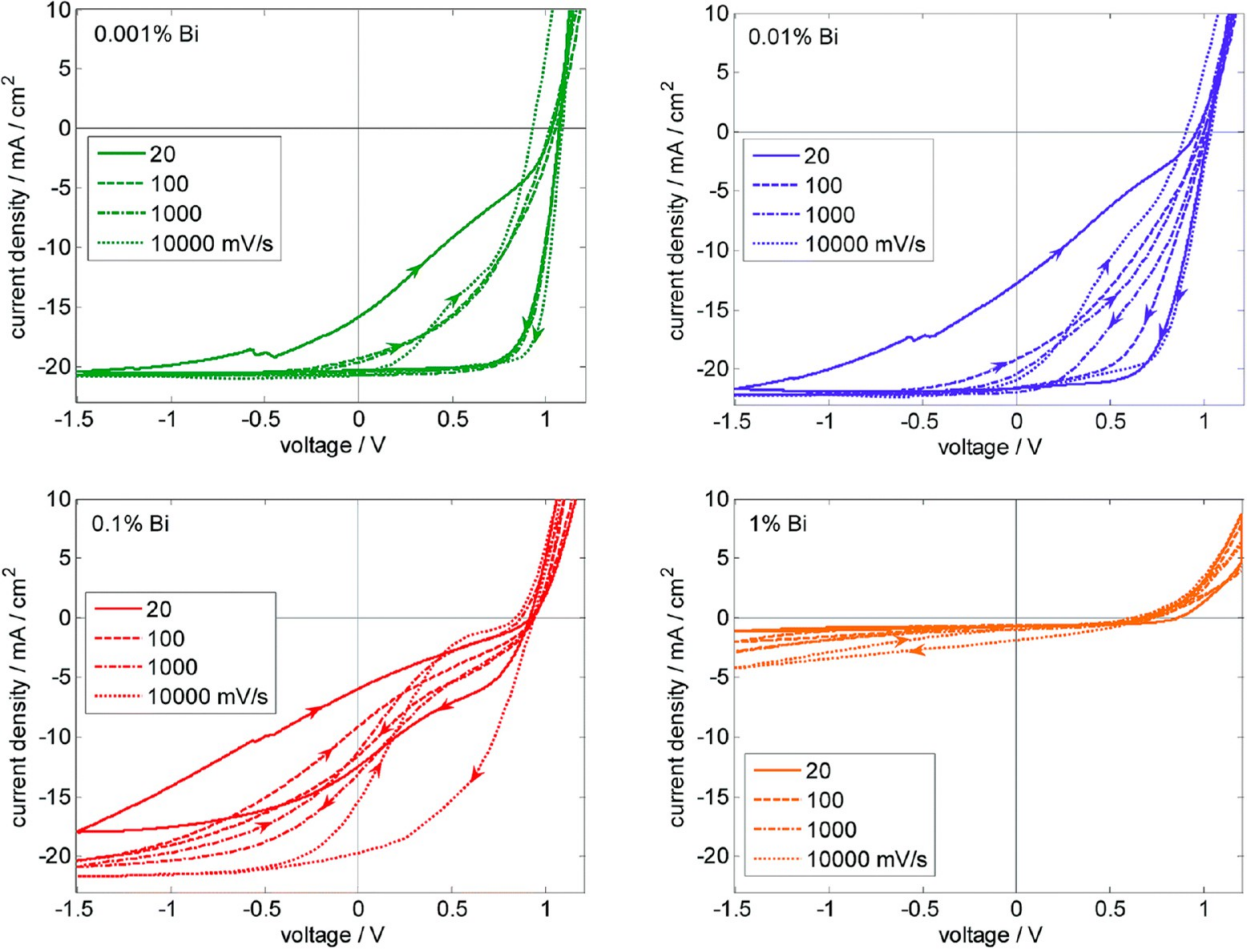
JV hysteresis loops starting from 1.2 V were measured at various voltage sweep rates measured at solar cells with different Bi concentrations. The reduced JSC and FF upon adding Bi are due to a strong dependence on charge extraction on the electric field. Adapted with permission from ref (20). Copyright 2019 Royal Society of Chemistry.

Transition metal doping is being extensively investigated to disseminate new optical, magnetic, and electronic properties on perovskite nanocrystals. For example, Liu and co-workers first reported Mn^2+^-doped CsPbCl_3_ with tunable bandgap and color.^142^ Ni^2+^ is another emerging dopant, though unlike Mn^2+^, it does not exhibit dual emission but decreases the perovskite crystal size and distribution with increasing Ni^2+^ concentration.^143^ Bi^3+^ doped lead halide perovskites (LHP) exhibit a broad (850- 1600 nm) NIR photoluminescence emission due to the dopant,^144^ but the host’s photoluminescence intensity decreases both with increasing Bi^3+^ concentration and over time. Also, the Bi^3+^ doped MAPbI_3_ solar cell performance dropped significantly in the presence of trace amounts of Bi^3+^. Figure 10 shows that with increasing Bi^3+^ amount the short circuit current density (Jsc) and the fill factor (FF) decrease. This is because Bi^3+^ generates deep and shallow electronic states near the bandgap and causes a reduction in charge carriers’ mobility and defective recombination.^20^ Elemental lead is a byproduct from the decomposition of PbI_2_ that accelerated the degradation of perovskite.^136^ Cr^2+^ has a high diffusion barrier but a low Cr_i_ (interstitial site) formation energy. Hence, the incorporation of Cr^2+^ in the MAPbI_3_ layer is kinetically hindered. Mo and W are the most compatible metals since they have higher Mo_i_ and W_i_ formation energies and diffusion barrier. Even though Cu and Ag can diffuse quickly into the MAPbI_3_ layer, they do not introduce any deep levels that cause nonradiative recombination. Meanwhile, Au trapped in the Pb vacancy causes significant nonradiative recombination. In contrast, Au, Ag, and Cu can easily diffuse in CsSnBr_3_, but, therein, Au does not cause nonradiative recombination.^24^ Lineva and co-workers showed that a small amount of LiBO_3_, LiPO_4_, and Bi_2_O_3_ doped in Li_0.33_La_0.57_TiO_3_ (LLTO) perovskite can downturn the sintering temperature.^17^ LiBO_3_ and LiPO_4_ present in LLTO raised the total conductivity by 1 order of magnitude due to the reduction of the Schottky barrier height.^145^ However, impurities (e.g., oxygen vacancy Sb, La, and K) in BaSnO_3_ perovskite decreased the thermal conductivity via different phonon scattering pathways, such as acoustic and low-frequency optical phonon scattering.^146^

In the case of MAPbI_3_ solar cells, noble and transition metal electrodes can introduce unintentional impurities and cause device degradation. Irrespective of the presence of a hole transport layer (HTL), it has been seen that some metal ions can diffuse into the absorption layer. Du and co-workers investigated the energy and kinetics of the electrode materials based on DFT calculations and found that the diffusion barrier is positively correlated with oxidation charge, a guideline for selecting a metal as an electrode in a solar cell.^13^ While point and intrinsic defects were being blamed for the inferior performance of MAPbI_3_-based solar cells, Liang and co-workers^147^ showed using DFT studies that atomic hydrogen interstitial, H_i_^+^, serves as an electrically active negative-U defect.^148^ But molecular hydrogen plays a chemically inert role. The H_i_^+^ defects can be easily incorporated from the reaction media, deprotonation of organic HTL in the solar cell, and the organic cations (e.g., MA^+^) in organic–inorganic hybrid perovskite. High-density H_i_^+^ in MAPbI_3_ can lead to poor solar cell performance due to polarization, hysteresis, and a photogenerated field-screening effect.

As removal of impurities may not always be practical or desirable, researchers have been considering the effect of impurities as part of experimental design and exploring creative ways to deal with them. Liang and co-workers^147^ used DFT calculations to predict the outcomes of atomic and molecular hydrogen in MAPbI_3_ and MASnI_3_ perovskites. They concluded that a slight increase in the amount of iodine or tin in the synthesis suppresses damage from atomic hydrogen. Similarly, Wang and co-workers^137^ studied the preparation of HTL using nickel oxide in a perovskite solar cell; they demonstrated that the efficiency and stability of the solar cell could be improved by preventing the absorption of nitrate ion, which is simply done by adding [BMIM]BF_4_ IL, since the resulting [BMIM]^+^ can preferentially absorb on the surface of Ni(OH)_2_.

## 7 Oxide Nanomaterials

Metal oxides have unique physical, chemical, electronic, and magnetic structures and properties compared with other nanomaterials. Their distinctive features have made them popular in diverse fields like sensors, magnetic imaging, photocatalysis, solar cell design, batteries, magnetic storage media, energy conversion, optoelectronics, and electronics.^149−154^ However, like other nanomaterials, impurities significantly impact the synthesis of metal oxide NPs. This section presents studies that encountered or sought evidence of impurities affecting the structure of metal oxide nanomaterials and their photocatalytic properties.

**Figure 11:**
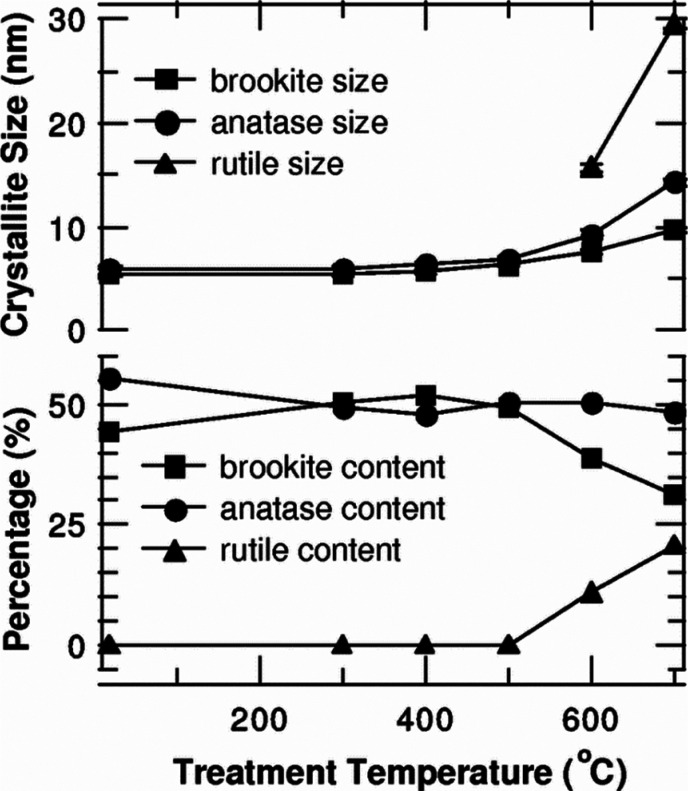
Crystallite size in TiO_2_ NPs as a function of the treatment temperature (top). Percentage of constituent structures as a function of treatment temperature (bottom). Adapted with permission from ref (15). Copyright 2007 American Physical Society.

Surface impurities affect the crystal growth and phase transformation of the NPs. Grena and co-workers used Car–Parrinello dynamics as a relaxation tool, combined with a standard DFT-based structural optimization method, to show the stabilizing effect of surface impurities on ZrO_2_ nanoclusters.^23^ When chemisorbed water was introduced as an impurity, it was observed that the clusters with greater impurity concentrations expressed more ordered and crystalline structures than those when impurities were absent. This ordering was proportional to the amount of water impurity. Thus, they proposed that the nanoclusters have an affinity to bind with impurities because the formation of the Zr–O bond is energetically favorable. These computational data are supported by the experimental data obtained from the X-ray total scattering pair distribution function (PDF). Another example is the well-reported phase transformation in TiO_2_ NPs that structurally incorporate impurities. Dopants with > +4 charge favor the anatase to rutile phase change, while dopants having < +4 charge slow the process.^155^ However, Y^3+^ is an exception that inhibits rutile transformation (Figure 11). Y^3+^ surface dopants increase the activation energy of anatase to rutile transformation by lowering the anatase surface energy of TiO_2_ NPs.^15^

Xiong and co-workers provided a fine correlation between particle size and impurities to the photoluminescence of ZnO NPs.^156^ ZnO NPs synthesized via a plasma synthesis technique have shown smaller particles to possess higher carboxylate (COO−) and hydroxyl (−OH) group impurities and vice versa. These impurities originated from the carbon-containing plasma species, and hydroxyl groups are present due to the hygroscopic nature of ZnO. These near-surface impurities serve as the nonradiative recombination centers or trapping centers, which leads to a decrease in the radiative recombination intensity for smaller-size particles.^157^ Annealing the samples in a vacuum can reduce the carboxylate and hydroxyl impurities, but that can increase the oxygen-deficient defects. Ga_2_O_3_ nanostructures synthesized without annealing tend to exhibit elevated interstitial impurities derived from oxygen atoms.^158^ Depending on the impurity concentration, the intensity of green emission can either decline or surge. As interstitial oxygen defects accumulate, there is an augmented electron population at the impurity level stemming from the transfer of electrons from the Ga_2_O_3_ conduction band. In another study, Calcabrini and co-workers showed fatty acid-stabilized oxide nanocrystal formation from metal nitrate precursor without adding any fatty acid to the reaction mixture.^159^ The authors discovered that metal nitrate precursors (e.g., Ce(NO_3_)_3_·6H_2_O) form complexes (e.g., [Ce-(RNH_2_)_n_(NO_3_)_3_]) upon reacting with OAm, the intended NP ligand. The nitrates then oxidize OAm to create fatty acid impurities which then become the primary capping ligands. The same phenomenon was observed during the preparation of the NiO, ZnO, and CeO_2-*x*_ NPs.

The doping of TiO_2_ has also been used to enhance its photocatalytic efficiency.^160,161^ For example, Al and Cu impurities decreased the bandgap value of TiO_2_ and increased its photocatalytic activity by trapping electrons and prolonging the recombination time of photogenerated electrons and holes.^162^ Xing and co-workers showed that Ti^3+^ self-doped TiO_2_ exhibits enhanced visible light absorption and photocatalytic efficiency without weakening UV-light photocatalytic activity.^163^ Furthermore, adsorbed chloride ions onto the TiO_2_ NP surface can also influence densification, phase transformation, and grain growth during the sintering of NPs.^7^ Impurities do not only affect the physical properties of the host nanocrystal; considering the kind of chemical reaction intended, the catalyst can be altered by selecting a particular doping material. For example, doping In_2_O_3_ nanocrystals with Bi atoms creates Lewis acidic/basic Bi^3+^–O^2–^ pairs which enhance the adsorption and activation of CO_2_ during photocatalytic hydrogenation.^164^ An important n-type semiconductor bismuth tungstate (Bi_2_WO_6_) has shown an increased nonlinear absorption upon La doping due to the charge separation efficiency of photogenerated charge carriers.^165^ Doping La causes contraction in the Bi_2_O_3_ lattice, and the defect sites present in the crystalline structure act as centers to reduce photogenerated electron–hole pair recombination. An enhanced nonlinear absorption has also been seen in nanostructures of Cr-doped NiO (Ni_0.98_Cr_0.02_O).^165^ The excellent nonlinear optical properties of the doped materials make them superior candidates for fabricating various optical devices. Impurities can also affect the localized surface plasmon resonance (LSPR) of semiconductor nanocrystals, as revealed by a systematic study using Sn, Ce, Ti, or Zr as a dopant in In_2_O_3_ nanocrystals. Tandon and co-workers reported that properties inherent to the dopant, like electropositivity, atomic radius, and stability of aliovalent oxidation state affect carrier concentration and damping but does not affect surface depletion, which seems to depend only on the characteristic of the In_2_O_3_ nanocrystal.^166^

## 8 Thermoelectric Materials

Thermoelectric materials can uniquely convert temperature differences into electrical energy or vice versa, making them a promising technology for a wide range of applications, from power generation to cooling and heating devices.^167−169^ However, the performance of thermoelectric materials is strongly influenced by the presence of impurities, which affect the electrical conductivity, thermal conductivity, and carrier concentration. Therefore, understanding the effect of impurities on thermoelectric materials is critical for optimizing their performance and developing more efficient and reliable thermoelectric devices. In this context, researchers are exploring various strategies, such as doping and nanostructuring, to mitigate the negative impact of impurities on thermoelectric materials, to improve their thermoelectric performance, and to widen their potential applications. This section presents an analysis of the theoretical effect of impurities on the properties of thermoelectric materials, followed by examples of the effects of oxygen or magnetic impurities and a summary of works seeking to improve thermoelectric materials by exploiting the role of impurities.

The figure of merit, 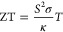
defines the efficiency of a thermoelectric material, where *S* is the Seebeck coefficient, σ is the electrical conductivity, κ is the thermal conductivity, and *T* is the absolute temperature.^169,170^ The higher the ZT value, the better the energy conversion efficiency. The impurities can change the density of states (DOS) around the materials’ Fermi level, which can alter the ZT value. Ionized impurities are charged atoms or molecules that have lost or gained one or more electrons, creating a charged defect in the material’s crystal lattice. When a carrier (either an electron or a hole) moves through the lattice, it can interact with the charged impurities and scatter off. This scattering process can affect the carrier mobility and, therefore, the material’s electrical conductivity. In the case of thermoelectric materials, ionized impurity scattering can also affect the Seebeck coefficient, which is a measure of the material’s ability to generate a voltage in response to a temperature difference. The Seebeck coefficient is related to the difference in energy between the Fermi level and the energy level of the carrier, and ionized impurities can shift the Fermi level, affecting the Seebeck coefficient.

Atmospheric oxygen can oxidize transition-metal-based thermoelectric materials to produce oxide impurities. Oxygen incorporation can degrade the crystalline quality of ScN film and increase the bandgaps from 2.2 to 3.1 eV. Higher oxygen contamination can also result in non-Arrhenius behavior of ScN film resistivity vs temperature, ultimately leading to a degenerate n-type conductivity.^171^ Thus, ScN fabrication requires a low oxygen environment or ultrahigh vacuum conditions. A reduction in the oxygen impurity under 3% in the reaction environment can decrease electrical resistivity 4-fold without affecting the Seebeck coefficient value of ScN film. This resulted in an estimated power factor of 3.2 Å ∼ 10^–3^ W m^–1^ K^–2^ at room temperature.^172^ The ZT of a material can be enhanced either by lowering the lattice thermal conductivity or by increasing the power factor S^2^·σ. Introducing impurities or dopants can change the DOS, the most traditional method to influence thermoelectric properties. Bi_2_Te_3_ demonstrates superior thermoelectric performance within the temperature range of 25–100 °C. Manzano and co-workers investigated how commercially available Te powder, and the impurities present can influence the material’s thermoelectric properties.^173^ Bi_2_Te_3_ film made with Te powder had alkali metal impurities (i.e., Li, K, and Na). These alkali metal ions acted as p-type dopants. On the other hand, Te powder with selenium impurity improved the Seebeck coefficient and the electrical properties of the film, behaving as an n-type dopant.

Magnetic elements, as dopants, can influence the figure of merit of Bi_2_Te_3_, Sb_2_Te_3_, and Bi_2_Se_3_ single crystals by changing the concentration of electrons and holes.^174^ Introducing magnetic impurities is a natural and effective method for exposing the topological properties of Dirac points such as linear dispersion,^175^ chirality,^176^ Berry phase,^177^ topological protection,^178^ and Dirac cones.^179^ Magnetic impurity doping of Bi_2_Te_3_, Sb_2_Te_3,_ and Bi_2_Se_3_ causes an increase in the effective scattering parameter r, indicating a shift in the primary electron (or hole) scattering mechanism from acoustic phonon scattering to impurity scattering in Fe- and Cr-doped samples. Doping with magnetic impurities causes a significant increase in the Seebeck coefficient for p-Bi_2-*x*_Fe_*x*_Te_3_ and Sb_2-*x*_Cr_*x*_Te_3_ (and a decrease for Bi_2-*x*_Fe_*x*_Se_3_), primarily due to changes in carrier concentration and Fermi energy level, and the heat conductivity decreases upon doping. This resulted in an enhancement in ZT value in n-Bi_2-*x*_Fe_*x*_Se_3_ (*T* < 100 K) and Sb_2-*x*_Cr_*x*_Te_3_ (*T* > 150 K). Other n-type Mg_3_Sb_2_-based materials have recently been reported to exhibit promising thermoelectric properties at a low- to midtemperature range; they are the competitive alternatives to conventional and environmentally harmful thermoelectric materials like Bi_2_Te_3_ or PbTe.^180−183^ These materials are regarded as p-type compounds due to the Mg-vacancies as calculated by Tamaki and co-workers.^183^ Electrical conductivity of Te-doped Mg_3_Sb_2_ exhibits an anomalous temperature dependency because of the same point defects arising from the Mg-vacancies.^184^ Therefore, it is crucial to control these point defects to enhance the thermoelectric properties, and that can be achieved by tuning the synthetic conditions like hot-pressing temperature and holding time. As described by Ren’s group,^182^ a sample of Mg_3.2_Sb_1.5_Bi_0.49_Te_0.01_ hot pressed at 1073 K (∼128 cm^2^ V^–1^ s^–2^) showed ∼228% increase in Hall mobility at room temperature than the sample prepared at 923 K (∼39 cm^2^ V^–1^ s^–2^). This leads to a dramatic enhancement in the power factor from ∼6 to ∼20 μW cm^–1^ K^–2^. There was an almost 4-fold increase in the room temperature electrical conductivity as well. Boosting carrier mobility (μ = , where *e* is the electron charge, *m* is the effective mass, and ⟨τ⟩ is the average relaxation time) can provide another route to improve thermoelectric properties, in addition to the conventional method of enhancing the Seebeck coefficient.^181^ The same group achieved an improved room temperature Hall mobility of ∼81 cm^2^ V^–1^ s^–1^ from ∼16 cm^2^ V^–1^ s^–1^ and a power factor of ∼13 μW cm^–1^ K^–2^ from ∼5 μW cm^–1^ K^–2^ by doping transition metal elements (i.e., Fe, Co, Hf, and Ta). This also decreased the thermal conductivity, and a ZT value of ∼1.7 was obtained in Mg_3.1_Co_0.1_Sb_1.5_Bi_0.49_Te_0.01_. In a different example, Ga doping in PbTe creates two types of impurity states, a shallow-level Ga^3+^ state, and a deep-level Ga^+^ state.^185^ This weakens the electron–phonon scattering, resulting in higher carrier mobility, and decreases lattice thermal conductivity.

Various strategies have been applied for ZT value improvement, defect engineering,^186−188^ band-engineering,^189,190^ nanostructuring,^191,192^ modulation doping,^193,194^ including resonant level,^195^ and ionized impurity scattering.^196^ All of these methods mentioned here can regulate the point defect in the lattice. However, dealing with line, planar, and bulk defects presents greater difficulties. These issues can be mitigated by utilizing a more precise and regulated synthetic approach at the NP level. Fiedler and co-workers suggested a technique for converting precisely defined powder into a compact inorganic thermoelectric material using solution processing to achieve specific features.^197^ Colloidal synthesis of thermoelectric materials in the presence of organic ligands will result in more controlled and organized microstructure formation.

## 9 Perspective and Conclusion

The presence of impurities is considered a problem in synthesis; even if adverse effects are not directly observed, the uncertain nature of impurities can lead to misinterpretation of the results. For this reason, efforts have been made to reduce or remove impurities. However, as doping has shown, impurities are not always avoidable or undesirable: they must simply be **known** and **controlled** for a synthesis to be considered successful and understandable.

As discussed herein, impurities influence the course of nanomaterial research worldwide. Attempts to work around impurities have bolstered our capability to decipher their role and invent creative ways to correct for their negative effects. Bridges between theoretical work and experimental design are working to account for the role of impurities and combat misinterpretation. Indeed, a major milestone toward alleviating this misinterpretation is the understanding that vendors prepare reagents in varying ways and with a diverse precursor lineup; this knowledge has tempered the expectation that nanoscience will be reproducible vendor-to-vendor and even lot-to-lot. Perhaps this discovery is disheartening to some, but the variability it imposes also increases the likelihood of new discoveries: how could we come to know that irradiation of Ag ions in the presence of acetate would yield nanoparticles without significant acetate impurities in PVAc?

The presence of impurities can even be exploited as a pseudointernal standard for the quantification of nanomaterials during synthesis and use.^198^ On the other hand, we know that mass purification of reagents to ultrapure levels can be impractical for scalability and sustainability. For example, the great demand for pure carbon in synthesizing carbon nanomaterials has forced an inquiry into more abundant carbon sources (though even the highest quality coal has up to 8% impurities).^199^ Instead, impurities are an omnipresent variable that researchers will need to work with, particularly as we move toward a more green and sustainable future.
